# PRRSV Infection Induces Gasdermin D-Driven Pyroptosis of Porcine Alveolar Macrophages through NLRP3 Inflammasome Activation

**DOI:** 10.1128/jvi.02127-21

**Published:** 2022-06-27

**Authors:** Sheng He, Lu Li, Haifan Chen, Xiaoli Hu, Wendi Wang, Hui Zhang, Ruiping Wei, Xiaoxiao Zhang, Yaosheng Chen, Xiaohong Liu

**Affiliations:** a State Key Laboratory of Biocontrol, School of Life Sciences, Sun Yat-sen University, Guangzhou Higher Education Mega Center, Guangzhou, Guangdong, People’s Republic of China; Cornell University

**Keywords:** porcine reproductive and respiratory syndrome virus, pyroptosis, NLRP3 inflammasome, dispersed *trans*-Golgi network, replication-transcription complex

## Abstract

For more than 3 decades, mounting evidence has associated porcine reproductive and respiratory syndrome virus (PRRSV) infection with late-term abortions and stillbirths in sows and respiratory disease in piglets, causing enormous economic losses to the global swine industry. However, to date, the underlying mechanisms of PRRSV-triggered cell death have not been well clarified, especially in the pulmonary inflammatory injury characterized by the massive release of pro-inflammatory factors. Here, we demonstrated that PRRSV infection triggered gasdermin D-mediated host pyroptosis *in vitro* and *in vivo*. Mechanistically, PRRSV infection triggered disassembly of the *trans*-Golgi network (TGN); the dispersed TGN then acted as a scaffold for NLRP3 activation through phosphatidylinositol-4-phosphate. In addition, PRRSV replication-transcription complex (RTC) formation stimulated TGN dispersion and pyroptotic cell death. Furthermore, our results indicated that TMEM41B, an endoplasmic reticulum (ER)-resident host protein, functioned as a crucial host factor in the formation of PRRSV RTC, which is surrounded by the intermediate filament network. Collectively, these findings uncover new insights into clinical features as previously unrecognized mechanisms for PRRSV-induced pathological effects, which may be conducive to providing treatment options for PRRSV-associated diseases and may be conserved during infection by other highly pathogenic viruses.

**IMPORTANCE** Porcine reproductive and respiratory syndrome virus (PRRSV) is one of the pathogens responsible for major economic losses in the global swine industry. Characterizing the detailed process by which PRRSV induces cell death pathways will help us better understand viral pathogenesis and provide implications for therapeutic intervention against PRRSV. Here, we showed that PRRSV infection induces GSDMD-driven host pyroptosis and IL-1β secretion through NOD-, LRR- and pyrin domain-containing protein 3 (NLRP3) inflammasome activation *in vitro* and *in vivo*. Furthermore, the molecular mechanisms of PRRSV-induced NLRP3 inflammasome activation and pyroptosis are elucidated here. The dispersed *trans*-Golgi network (TGN) induced by PRRSV serves as a scaffold for NLRP3 aggregation into multiple puncta via phosphatidylinositol 4-phosphate (PtdIns4P). Moreover, the formation of PRRSV replication-transcription complex is essential for TGN dispersion and host pyroptosis. This research advances our understanding of the PRRSV-mediated inflammatory response and cell death pathways, paving the way for the development of effective treatments for PRRSV diseases.

## INTRODUCTION

Porcine reproductive and respiratory syndrome virus (PRRSV) is an enveloped, single-stranded, positive-sense RNA virus with a genome of about 15 kb that belongs to the family *Arteriviridae* in the order *Nidovirales* (1). PRRSV causes porcine reproductive and respiratory syndrome (PRRS), which is a huge threat to the global swine industry (2, 3). PRRS is characterized by reproductive failure of sows and respiratory diseases of piglets and growing pigs, and it can predispose pigs to secondary bacterial infections (4, 5). Several attempts have been made to investigate the basic biology of PRRSV, ranging from genetic characteristics to porcine immune response (1, 4, 5). Nonetheless, PRRSV infection has not been well controlled since the first outbreak in the late 1980s (5, 6).

PRRSV has previously been proven to induce cell apoptosis after infection both *in vitro* and *in vivo* (6–8). Since apoptotic cells are generally immune-silent (9), numerous studies have revealed a contentious subject: PRRSV infection promotes an inflammatory response in infected cells and inflammatory damage to lung tissue. Highly pathogenic PRRSV infection induces secretion of the pro-inflammatory factor HMGB1 and interleukin (IL)-1β, which are indicators of pyroptosis (10–12). Therefore, in addition to apoptosis, it is hypothesized that PRRSV infection may also cause pyroptosis, which is defined as gasdermin-mediated programmed cell necrosis (12, 13). Our understanding of the pathological damage caused by PRRSV infection is inadequate, and its pathological mechanisms warrant further investigation for the development of effective vaccines and antiviral drugs.

Pro-caspase-1, ASC (apoptosis-associated speck-like protein containing a CARD), and inflammasome nucleators such as NOD-like receptors (NLRs), AIM2, and pyrin comprise the cytoplasmic multi-protein complex known as inflammasomes (14). Among these, the NOD-, LRR- and pyrin domain-containing protein 3 (NLRP3) inflammasome acts as a classic pyroptosis mediator that can be activated by various types of viral infections, including Zika, influenza A (IAV), and hepatitis C viruses (HCV) (15–18). The NLRP3 inflammasome can be activated by various stimuli. Apart from viral proteins, numerous environmental irritants and endogenous metabolic products which act as damage-associated signals can also activate the NLRP3 inflammasome (19–21). Notably, it has been proven that the unifying feature of multiple NLRP3 inflammasome-activating stimuli is that they disrupt *trans*-Golgi network (TGN) structure, resulting in a dispersed TGN (dTGN) (22, 23). The dTGN serves as a scaffold for NLRP3 aggregation via phosphatidylinositol-4-phosphate (PtdIns4P) and then oligomerizes with ASC to form inflammasome (23).

Once the NLRP3 inflammasome is assembled, it serves as a platform for autocleavage of pro-caspase-1 to yield active capsase-1 (CASP1) (24). In addition to converting the inactive pro-inflammatory cytokines IL-1β and IL-18 into their biologically mature forms, the activated CASP1 attaches to the C terminus of gasdermin D (GSDMD) and cleaves the tetrapeptide after GSDMD D275. The free N terminus of GSDMD attaches to lipids and oligomerizes spontaneously, causing cell content leakage and eventually cell death, termed pyroptosis (24). Although pyroptosis serves as a defensive mechanism to restrict pathogen replication and spreading, activating this cell death pathway has potentially deleterious hyper-inflammatory consequences in host tissues (25). Moreover, GSDMD can be hijacked by viral proteins to prevent host cell pyroptosis. For example, GSDMD is cleaved between Q193 and G194 by enterovirus 71 3C, leaving a shortened GSDMD N segment that is unable to generate pores on membranes (26). Recently, *in vitro* experiments have suggested that PRRSV infection enhances IL-1β production via NLRP3 inflammasome activation (27, 28), but whether PRRSV infection can assemble NLRP3 inflammasome and ultimately lead to pyroptosis *in vivo* remains unknown. In addition, the molecular mechanisms of PRRSV-induced inflammasome activation and pyroptosis still need a further survey.

In this study, we sought to fill this gap in our knowledge by conducting PRRSV challenge experiments *in vitro* and *in vivo* to investigate whether viral infection induces pyroptosis and its molecular mechanism. Cellular and clinical sample studies revealed that PRRSV infection triggers GSDMD-dependent pyroptosis via NLRP3 inflammasome assembly, which is characterized by lactate dehydrogenase (LDH) release and IL-1β secretion. Further mechanistic investigation demonstrated that PRRSV infection disassembles the TGN into dTGN. The dTGN served as a scaffold for NLRP3 aggregation into multiple puncta via PtdIns4P. Meanwhile, knockdown of TMEM41B, an endoplasmic reticulum (ER)-resident host protein, restricts PRRSV replication-transcription complex (RTC) formation, maintains the morphology of TGN, and abolishes the occurrence of pyroptosis. Together, our results demonstrate that in addition to apoptosis, PRRSV infection causes host pyroptosis *in vitro* and *in vivo*, and they elucidate its mechanism. These findings should aid in the exploration of the underlying processes of PRRSV pathogenesis and provide vital insights for the development of effective therapies against PRRSV infection.

## RESULTS

### PAMs and Marc-145 cells undergo pyroptotic cell death in response to PRRSV infection.

Pyroptosis is a form of programmed cell death triggered by perturbations of extracellular or intracellular homeostasis upon pathogen invasion, which manifest as inflammasome formation, caspase activation, gasdermin D cleavage, and plasma membrane damage; and hence, it mediates LDH release and IL-1β secretion (12).

**FIG 1:**
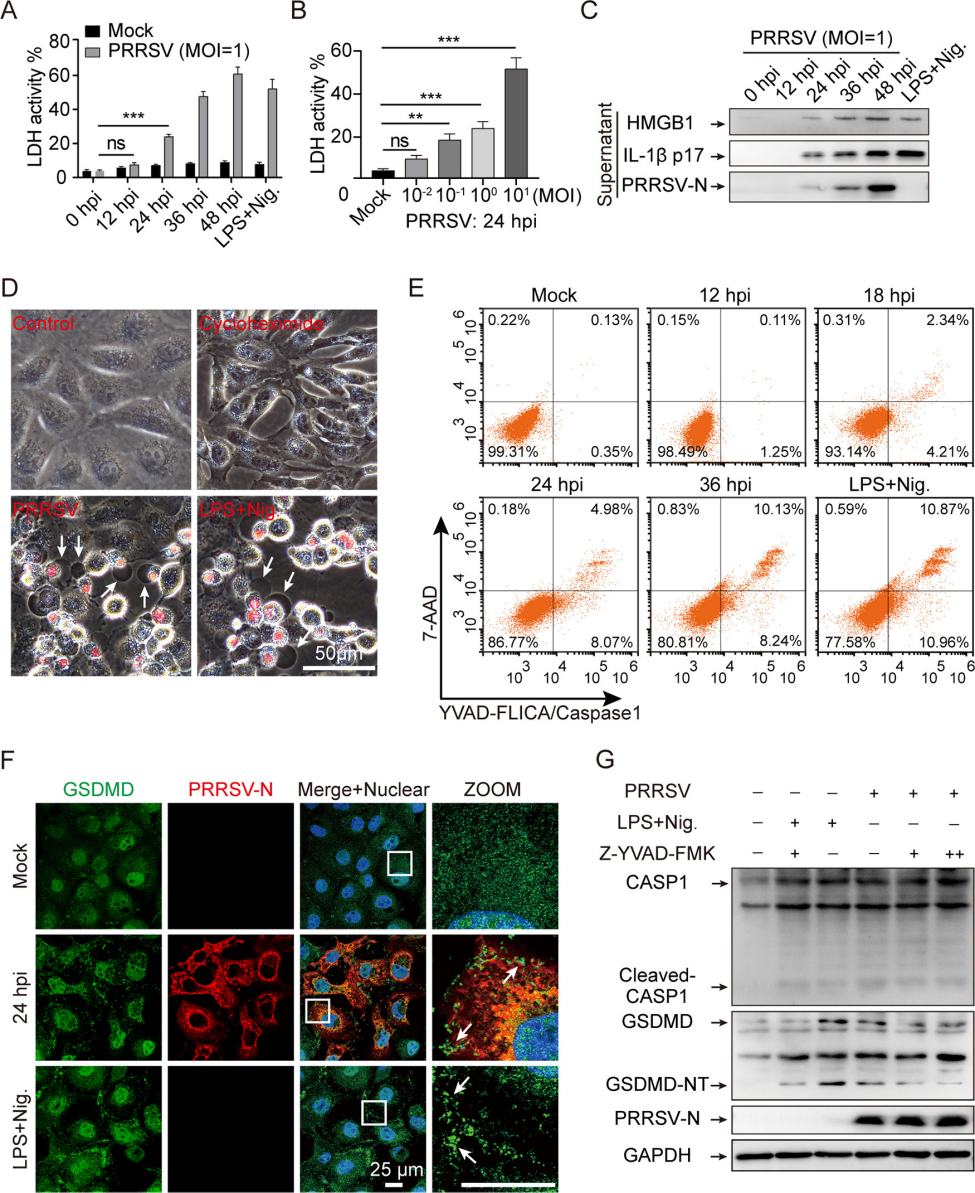
Porcine reproductive and respiratory syndrome virus (PRRSV) infection induces pyroptosis in porcine alveolar macrophages (PAMs) and Marc-145 cells. (A to C) PRRSV infection induces pyroptosis patterns in PAMs. PAMs were infected with PRRSV at different times or at various multiplicities of infection (MOIs). The positive control for pyroptosis was lipopolysaccharide (LPS, 100 ng/mL)-primed for 6 h and stimulated with nigericin (10 μM) for 2 h. Lactate dehydrogenase (LDH) assay in supernatants from PAMs at different times (A) and at various MOIs (B). LDH in the supernatants after complete lysis of the target cells by the lysis solution was taken as 100%. (C) HMGB1, IL-1β, and PRRSV-N in culture supernatants were determined by Western blotting. (D) Phase-contrast images of Marc-145 cells were treated with cycloheximide (CHX, 50 μM, apoptotic drug) or nigericin (10 μM, pyroptotic drug) or infected with PRRSV for 36 h, and stained with 7-aminoactinomycin D (7-AAD). Arrows indicate pyroptotic cells. (E) Flow cytometry analysis of FAM-YVAD-FMK FLICA- and 7-AAD-stained cells. (F) An immunofluorescence assay confirmed the alterations in gasdermin D (GSDMD) distribution in Marc-145 cells following PRRSV infection or LPS/nigericin treatment. Cells were stained with GSDMD (green) and PRRSV-N (red) antibodies and analyzed by confocal microscopy. Nuclei were visualized by staining with DAPI (4’,6-diamidino-2-phenylindole). Arrows indicate GSDMD dots. (G) Z-YVAD-FMK attenuates PAM pyroptosis after PRRSV infection. PAMs were preincubated with 5 or 20 μM Z-YVAD-FMK or an equal volume of dimethyl sulfoxide (DMSO) for 1 h, then infected with PRRSV for 24 h or exposed to LPS and nigericin as previously described (A). GSDMD cleavage was detected by Western blotting. Glyceraldehyde-3-phosphate dehydrogenase (GAPDH) is shown as an internal control. GSDMD-NT, the N-terminal cleavage product of GSDMD. Data are means ± SEM of triplicate samples and are representative of three independent experiments. Significant differences are indicated as follows: **, *P* < 0.01 and ***, *P* < 0.001 (one-way analysis of variance [ANOVA]). LPS+Nig., LPS + nigericin; ns, not significant.

To determine whether PRRSV infection provokes pyroptosis, during PRRSV infection, we measured lactate dehydrogenase activity in porcine alveolar macrophage (PAM) culture supernatants, which indicated extravasation of cellular contents. With the prolongation of PRRSV infection, compared to the mock infection, the amount of LDH in the culture supernatants of PAMs gradually increased at 24 h postinfection (hpi), which was consistent with the positive control for pyroptosis (Fig. 1A). Meanwhile, LDH was also released in a multiplicity of infection (MOI)-dependent manner (Fig. 1B), indicating the loss of membrane integrity during PRRSV infection. Western blot analysis demonstrated that the mature processed form of IL-1β, p17 subunit, and HMGB1 were secreted into the supernatant (Fig. 1C). Despite the absence of secreted signal peptides, mature IL-1β and HMGB1 appeared in cell culture supernatants, indicating that the cell membrane permeability was altered and caspase-1 was activated (9). To better understand the potential link between PRRSV infection and cell membrane impairment, we then tested the biological effect of PRRSV-induced pyroptosis on cultured Marc-145 cells that were fully permissive to PRRSV replication *in vitro*. Based on the morphological examination, we found typical pyroptotic changes, along with cell swelling, membrane rupture, and staining with a small membrane-impermeable DNA intercalator, 7-aminoactinomycin D (7-AAD), which was consistent with the lipopolysaccharide (LPS)/nigericin-induced pyroptosis control group but not with the mock control or the cycloheximide (CHX)-induced apoptosis control (Fig. 1D). These findings suggest that PRRSV-induced host pyroptosis is responsible for the loss of plasma membrane integrity.

Moreover, we used the fluorescent probe FAM-YVAD-FMK FLICA/7-AAD double-staining assay to investigate the relationship between CASP1 activation and cell membrane damage during PRRSV infection. PAMs were infected with PRRSV for the indicated periods, and then cells were incubated with FAM-YVAD-FMK FLICA for 1 h and with 7-AAD for another 10 min, allowing the fluorescent probe to enter the cell and irreversibly bind to activated CASP1. Fluorescently labeled PAMs were then detected by flow cytometry, and it was found that CASP1 activation occurred before cell membrane damage and that the proportion of double-positive cells increased as the duration of PRRSV infection increased (Fig. 1E). These findings suggested that CASP1 activation was linked to cell membrane damage. In addition, GSDMD was diffusely distributed in the nucleus and cytoplasm of mock-infected cells, but it aggregated into dots and distributed near the cell membrane in the presence of PRRSV infection and nigericin treatment (Fig. 1F). The GSDMD spliceosome is an executive protein which is responsible for pyroptosis (12). We carried out an immunoblotting assay to determine whether PRRSV infection can induce GSDMD cleavage. Compared to that in the uninfected control, the N-terminal cleavage fragments of GSDMD (GSDMD-NT) were detected after PRRSV infection, suggesting that PRRSV infection triggered GSDMD cleavage in PAMs (Fig. 1G). Furthermore, the GSDMD cleavage was dramatically restrained by Z-YVAD-FMK, a CASP1-specific inhibitor, without affecting viral replication, implicating that CASP1 mediates GSDMD cleavage in PRRSV-infected cells (Fig. 1G). Collectively, PAMs and Marc-145 cells undergo pyroptosis as a result of activated CASP1-induced GSDMD cleavage during PRRSV infection.

### PRRSV infection induces PAM pyroptosis *in vivo*.

**FIG 2:**
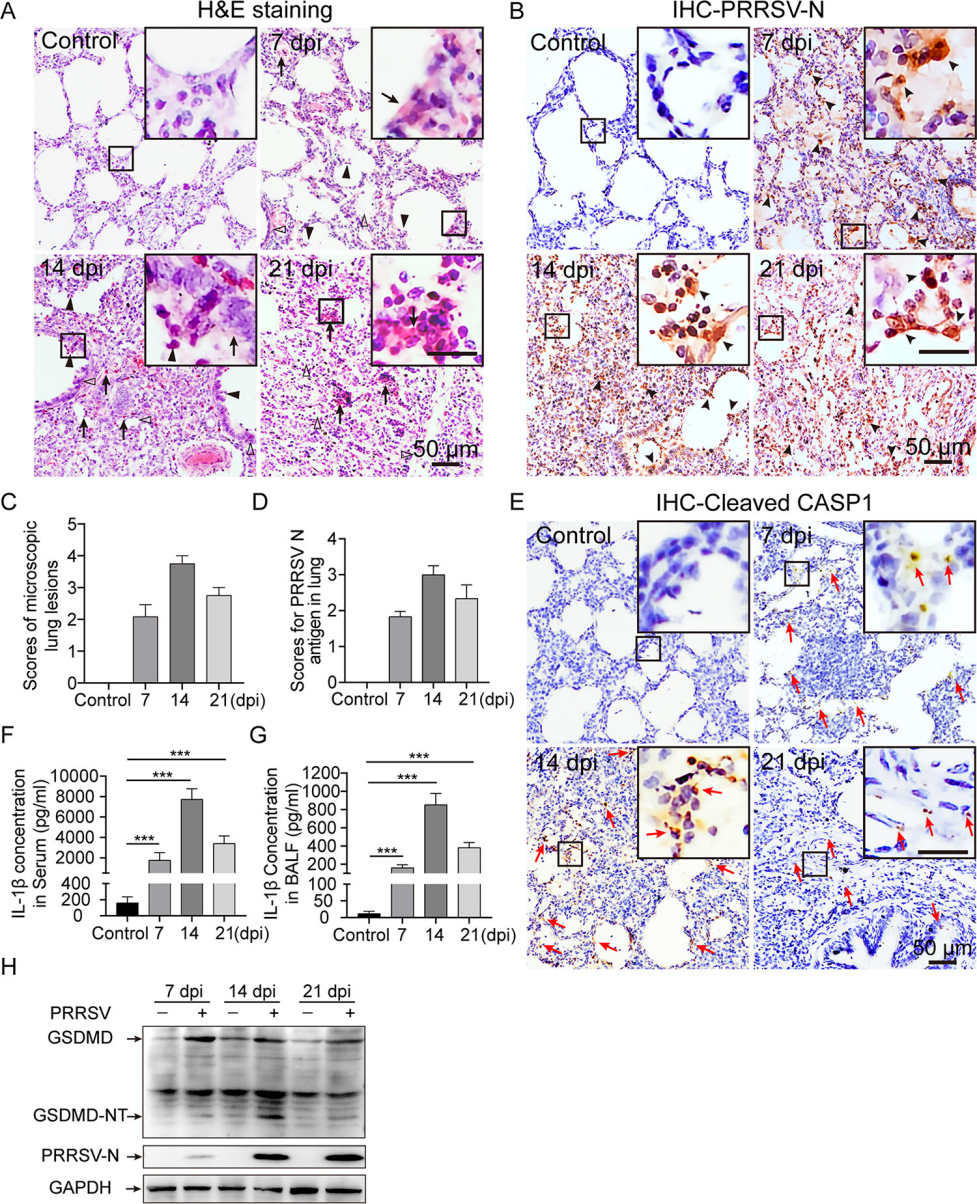
PRRSV infection causes PAM pyroptosis *in vivo*. (A) Representative micrographs of lungs obtained from mock- and PRRSV-infected piglets (7, 14, and 21 days postinfection [dpi]) showing histopathological changes. Lung sections were stained with hematoxylin and eosin (H&E). Solid and thin arrows indicate hemorrhage and hyperemia or infiltration of inflammatory cells within alveolar spaces and alveolar septum. Triangle indicates interlobular septal thickening, inflammatory cell infiltration around the bronchiole, or narrowed alveolar spaces. Solid triangle indicates inflammatory cells, necrotic debris, and exfoliated epithelial cell infiltrate in the alveolar and bronchiole. No significant changes in the lungs of mock-infected pigs. (B) Immunohistochemistry (IHC) was used to examine the PRRSV-N antigen in piglet lung tissues, using anti-PRRSV-N antibody. Arrowhead indicates PRRSV-N antigen signals in macrophages located within or around alveolus and bronchus. (C and D) Scores of microscopic lung lesions and PRRSV-N antigen for piglets infected with PRRSV. (E) Immunohistochemistry was used to examine the activated capsase-1 (CASP1) in piglet lung tissues, using anti-cleaved-CASP1 antibody. Red arrow indicates cleaved-CASP1 signals. (F and G) Enzyme-linked immunosorbent assay (ELISA) was used to measure IL-1β levels in serum and bronchoalveolar lavage fluid (BALF) from mock- and PRRSV-infected piglets. (H) Immunoblotting analysis was used to assess GSDMD cleavage in lung tissues of piglets with and without PRRSV infection. GSDMD-N is the N-terminal cleavage product of GSDMD. Significant differences from mock-infected piglets are shown as ***, *P* < 0.001 (one-way ANOVA). Data are representative of three experiments (mean ± SEM of *n* = 6 or *n* = 3).

PRRSV challenge (GDBY1 strain) was performed to confirm whether PRRSV infection could trigger pyroptosis *in vivo*. Microscopic lesions in lungs were observed, and immunohistochemistry was applied to examine PRRSV antigens in lungs. Compared with that in the mock-infected control group, severe inflammatory damage appeared in the PRRSV-infected lungs at the indicated time points. Histopathological changes in PRRSV-infected lungs were characterized by inflammatory cell infiltration, interstitial pneumonia, hyperemia, dropout of alveolar epithelial cells, alveolar spaces narrowed until the lung structure disappeared, and necrotic debris infiltration within the alveolar spaces (Fig. 2A). Furthermore, immunohistochemical staining exhibited that PRRSV-infected lungs showed strong PRRSV-N positive signals, which were mainly found in macrophages surrounding the bronchia, bronchiole, and alveolar septum (Fig. 2B). The average histopathological and immunohistochemical scores of the PRRSV-infected group were significantly elevated, whereas the mock-infected control group had no obvious microscopic lesions and positive PRRSV-N signals (Fig. 2C and D).

Immunohistochemical experiments on piglet lung tissues using cleaved CASP1 antibody revealed that mock-infected piglet lung showed negative activated CASP1 signals, whereas PRRSV-infected piglet lungs exhibited abundant cells with activated CASP1 signals (Fig. 2E). Moreover, compared with the mock-infected control group, it was remarkable that the concentration of IL-1β in serum and bronchoalveolar lavage fluid was significant increased in virus-infected piglets (Fig. 2F and G). In addition, our results showed that significant GSDMD cleavage occurred in the PRRSV-infected piglet lungs compared with that in the mock-infected groups (Fig. 2H). In summary, our results demonstrate that PRRSV infection triggers pyroptotic cell death *in vivo*.

### Pyroptosis is caused by NLRP3-dependent GSDMD cleavage during PRRSV infection.

**FIG 3:**
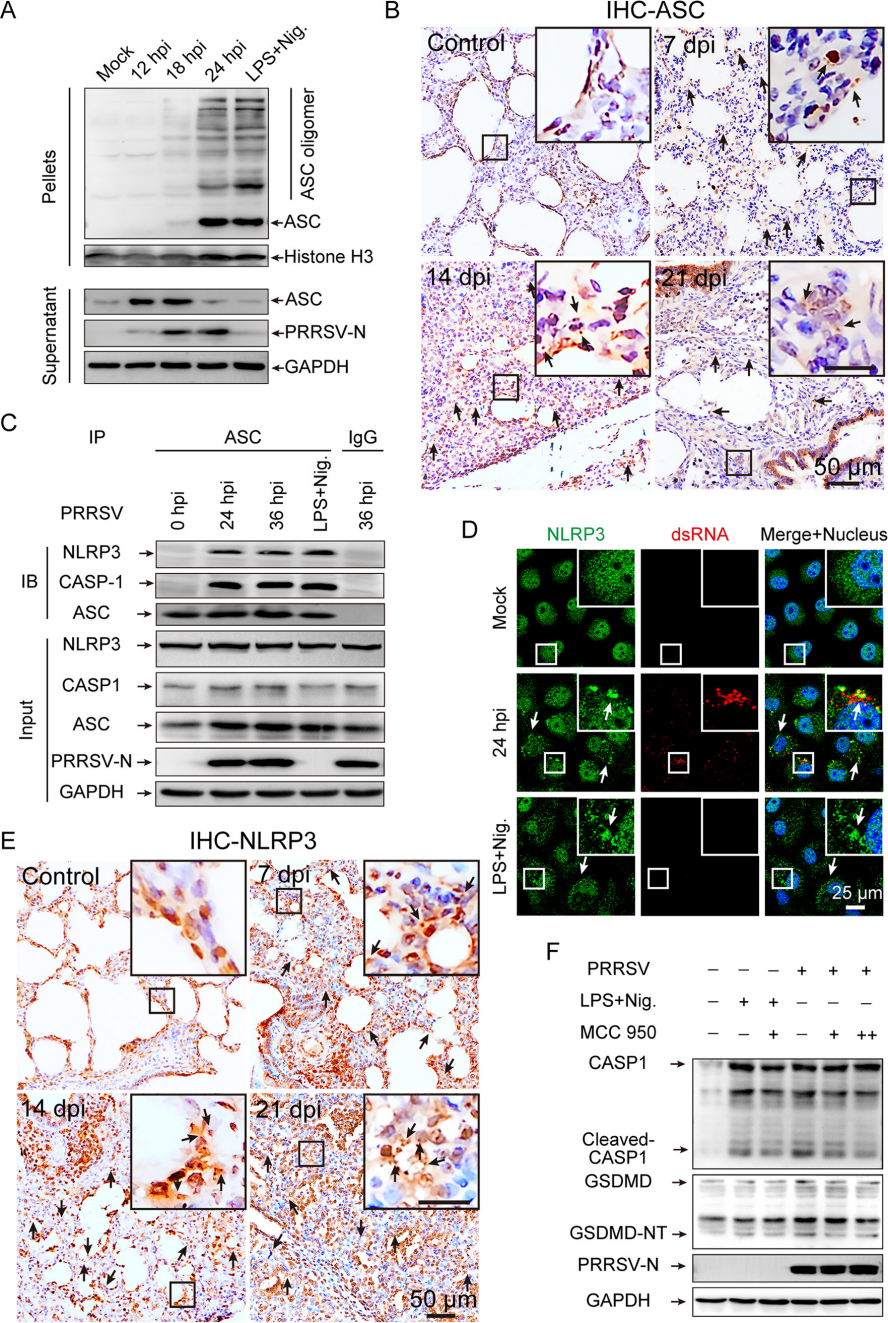
NOD-, LRR- and pyrin domain-containing protein 3 (NLRP3)-dependent GSDMD cleavage provokes pyroptosis in PRRSV infection. (A) Cell lysates were prepared and the pellets were subjected to ASC (apoptosis-associated speck-like protein containing a CARD) oligomerization assays (top panel). ASC and PRRSV-N in the supernatants were determined by Western blotting, with GAPDH as an internal control of the lysate supernatants (bottom row). (B) Representative images of ASC immunohistochemical staining in lung sections from 6-week-old pigs. Arrows indicate ASC specks. (C) PAMs were infected with PRRSV (MOI = 1) at the indicated times or administered LPS for 6 h followed by nigericin for 1 h as a positive control. Anti-ASC antibody or isotype control IgG was used for immunoprecipitation, followed by immunoblotting for NLRP3, CASP1, and ASC. Results are representative of two individual experiments. (D) A representative image of Marc-145 cells containing NLRP3 aggregates (green). Marc-145 cells were infected with PRRSV (MOI = 1) for 24 h or treated with LPS for 12 h followed by nigericin for 1 h as a positive control. Subcellular localizations of NLRP3 (green) and dsRNA (red) and the nucleus marker DAPI (blue) were examined under confocal microscopy. Inset (white) shows a higher magnification of a cell containing NLRP3 puncta. (E) Representative images of NLRP3 immunohistochemical staining in lung sections from 6-week-old pigs. Arrows indicate NLRP3 aggregates. (F) PAMs were pretreated with MCC950 for 1h before being infected with PRRSV (MOI = 1) for 24 h or stimulated with LPS and nigericin. Cleaved GSDMD and activated CASP1 in cell lysates were analyzed by Western blotting using the indicated antibodies. GAPDH is shown as an internal control. MCC950 concentrations of 5 and 20 μM are indicated by “+” and “++,” respectively. Data are representative of the results of three independent experiments.

We further sought to investigate the cellular mechanisms by which PRRSV induces pyroptosis. As reported by Lamkanfi and Dixit (29), the oligomerization of ASC is a direct indicator of inflammasome activation. The effect of PRRSV infection on ASC oligomerization was verified in PAMs, which were treated with LPS for 6 h followed by nigericin for 1 h as the positive control. By ASC oligomerization assays, prominent oligomerization of ASC was observed between 18 and 24 h after PRRSV infection, and no ASC oligomerization was observed in mock-infected PAMs (Fig. 3A). Furthermore, the ability of PRRSV infection to form ASC specks was assessed *in vivo* with immunohistochemical experiments utilizing the ASC-specific antibody on lung tissue sections. In contrast to that in mock-infected piglet lung tissue sections, histological analyses displayed that PRRSV facilitated ASC oligomerization in the lung tissues of infected piglets (Fig. 3B). Collectively, these results support the idea that ASC oligomerization is involved in the formation of the PRRSV-activated inflammasome.

Thus, we have shown that the inflammasome formed by the PRRSV infection is ASC-dependent. As RNA virus infections classically induce the inflammasome in an NLRP3-dependent manner, we set out to determine whether PRRSV infection activates the NLRP3 inflammasome (16, 30). We performed co-immunoprecipitation (Co-IP) assays to investigate the presence of endogenous NLRP3-ASC-CASP1 complexes in PAMs by anti-ASC antibody or isotype control IgG. NLRP3-ASC-CASP1 complexes were obviously detected by immunoprecipitation with anti-ASC antibody in PRRSV-infected and LPS/nigericin-stimulated PAMs, whereas immunoprecipitation with isotype control IgG did not detect any (Fig. 3C). Additionally, neither NLRP3 nor CASP1 was coprecipitated with ASC in mock-infected PAMs (Fig. 3C). Otherwise, NLRP3 redistributed from a diffused expression pattern into distinct, bright aggregates after 24 h of PRRSV infection, as well as in the LPS/nigericin stimulation Marc-145 cells, indicating NLRP3 inflammasome complex formation (Fig. 3D). Interestingly, immunohistochemistry of lung tissue sections showed that NLRP3 formed distinct small aggregates in PRRSV-infected piglets, while it was diffusely distributed in both the nucleus and cytosol of mock-infected piglets, supporting the conclusion that PRRSV infection can induce inflammasome activation in an NLRP3-dependent manner *in vivo* (Fig. 3E). To further demonstrate that NLRP3 inflammasome is mainly responsible for mediating the pyroptosis induced by PRRSV infection, PAMs were treated with MCC950 (the exclusive inhibitor of NLRP3) prior to PRRSV infection and nigericin stimulation (31). It was shown that both CASP1 activation and GSDMD cleavage were significantly reduced by MCC950 treatment after PRRSV infection in the positive control, whereas the expression of pro-CASP1 and full-length GSDMD was not affected in cell lysates (Fig. 3F). Therefore, these results demonstrate that PRRSV infection activates NLRP3 inflammasome to induce pyroptosis *in vitro* and *in vivo*.

### PRRSV replication or protein production is required for NLRP3 inflammasome activation and pyroptosis.

**FIG 4:**
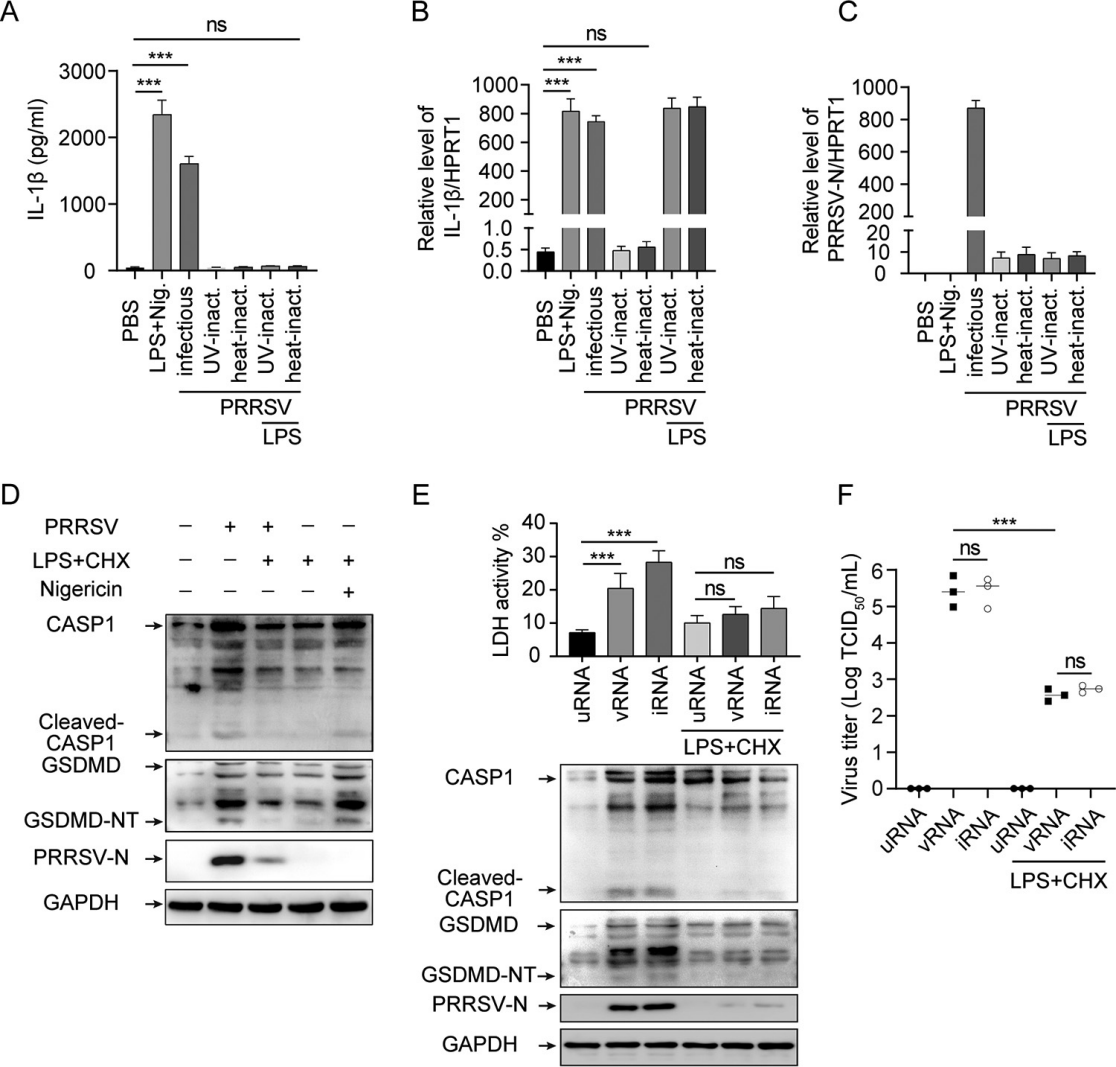
PRRSV replication or protein production is required to induce pyroptotic cell death. (A to C) PAMs were treated with LPS for 6 h and then stimulated with nigericin for 2 h, infected with PRRSV (MOI = 1) for 24 h, or inoculated with heat-inactivated or UV-inactivated PRRSV for 24 h in the presence or absence of LPS. IL-1β levels in the supernatants were determined by ELISA (A). Intracellular mRNA levels of IL-1β (B) and PRRSV-N (C) were measured using real-time quantitative reverse transcriptase PCR (qRT-PCR). (D) PAMs were inoculated with PRRSV for 2 h before being treated with CHX (50 μM) or DMSO, with the eventual infection lasting for 24 h; or the were primed with LPS for 6 h, treated with CHX (50 μM) for 1 h, and then treated with nigericin. Cleaved CASP1 and GSDMD (GSDMD-NT), PRRSV-N, and GAPDH levels in cell lysates were determined by Western blotting. (E and F) Marc-145 cells were transfected with 20 μg of total RNA from uninfected cells (uRNA), PRRSV genomic RNA (vRNA), or total RNA from PRRSV-infected cells (iRNA) for 24 h in the presence or absence of CHX. Activated CASP1, cleaved GSDMD, PRRSV-N, and GAPDH levels in cell lysates were determined by Western blotting (E). The percentage of cells undergoing pyroptosis was measured by analyzing the levels of released LDH in the supernatants. (F) Virus titers in the supernatants of the cells examined in panel E. Significant differences are shown as ***, *P* < 0.001 (one-way ANOVA). ns, not significance. Values represent mean ± SEM (*n* = 3). Results are representative of at least three separate experiments.

We evaluated the effects of PRRSV replication, genomic RNA, viral RNA transcripts, and viral proteins on the activation of pyroptosis. Under the LPS-treated conditions, UV-inactivated (UV-inact.) and heat-inactivated (heat-inact.) PRRSV failed to induce IL-1β secretion from PAMs, while infectious virions induced IL-1β secretion from PAMs (Fig. 4A). Moreover, both the IL-1β mRNA expression level and PRRSV RNA were significantly increased after PRRSV infection, but not after UV- or heat-inactivated PRRSV treatment (Fig. 4B and C). These results suggest that PRRSV replication is required for the activation of IL-1β. In addition, in the presence of cycloheximide (CHX, a translation inhibitor), pyroptosis induced by PRRSV infection was intensively inhibited, indicating that suppression of PRRSV replication or virus-derived proteins translation relieves pyroptosis symptoms, including decreased CASP1 activation and GSDMD cleavage (Fig. 4D). Notably, CHX was not able to prevent pyroptosis from extracellular nigericin stimulation (Fig. 4D), which is consistent with a previous report, as extracellular nigericin stimulation directly activates NLRP3 inflammasome through the formation of dTGN (23). In other words, *de novo* translation is not required for nigericin-induced NLRP3 inflammasome activation. These results suggest that activation of pyroptosis requires PRRSV replication or virus-derived protein translation rather than PRRSV genomic RNA.

The hypothesis that the pyroptosis resulting from PRRSV infection is due to PRRSV replication or virus-derived protein translation was also supported by the assays in Marc-145 cells. We compared the ability of PRRSV genomic RNA (vRNA) and PRRSV RNA transcripts (iRNA) to activate the NLRP3 inflammasome after transfection by Lipofectamine 3000, under the condition that total RNA from uninfected cells (uRNA) served as a negative control. Transfection with vRNA or iRNA induced caspase-1 activation, GSDMD cleavage, and LDH release (Fig. 4E), indicating that the NLRP3 inflammasome and pyroptosis were activated. However, in the presence of CHX, both of them failed to elicit NLRP3 inflammasome activation and subsequent pyroptosis, revealing that unlike in IAV infection (32), PRRSV genomic RNA and viral RNA transcripts do not activate NLRP3 (Fig. 4E). Intriguingly, since PRRSV is a positive single-stranded RNA virus, transfection of Marc-145 cells with vRNA and iRNA at 24 h posttransfection resulted in virion production in the supernatant. However, in the presence of CHX treatment, the production of virions was severely inhibited as measured by 50% tissue culture infective dose (TCID_50_) (Fig. 4F). Collectively, PRRSV replication or viral protein translation, rather than viral genomic RNA and viral RNA transcripts, mediates NLRP3 inflammasome activation and pyroptosis.

### PtdIns4P on dispersed *trans*-Golgi network mediates NLRP3 inflammasome activation during PRRSV infection.

**FIG 5:**
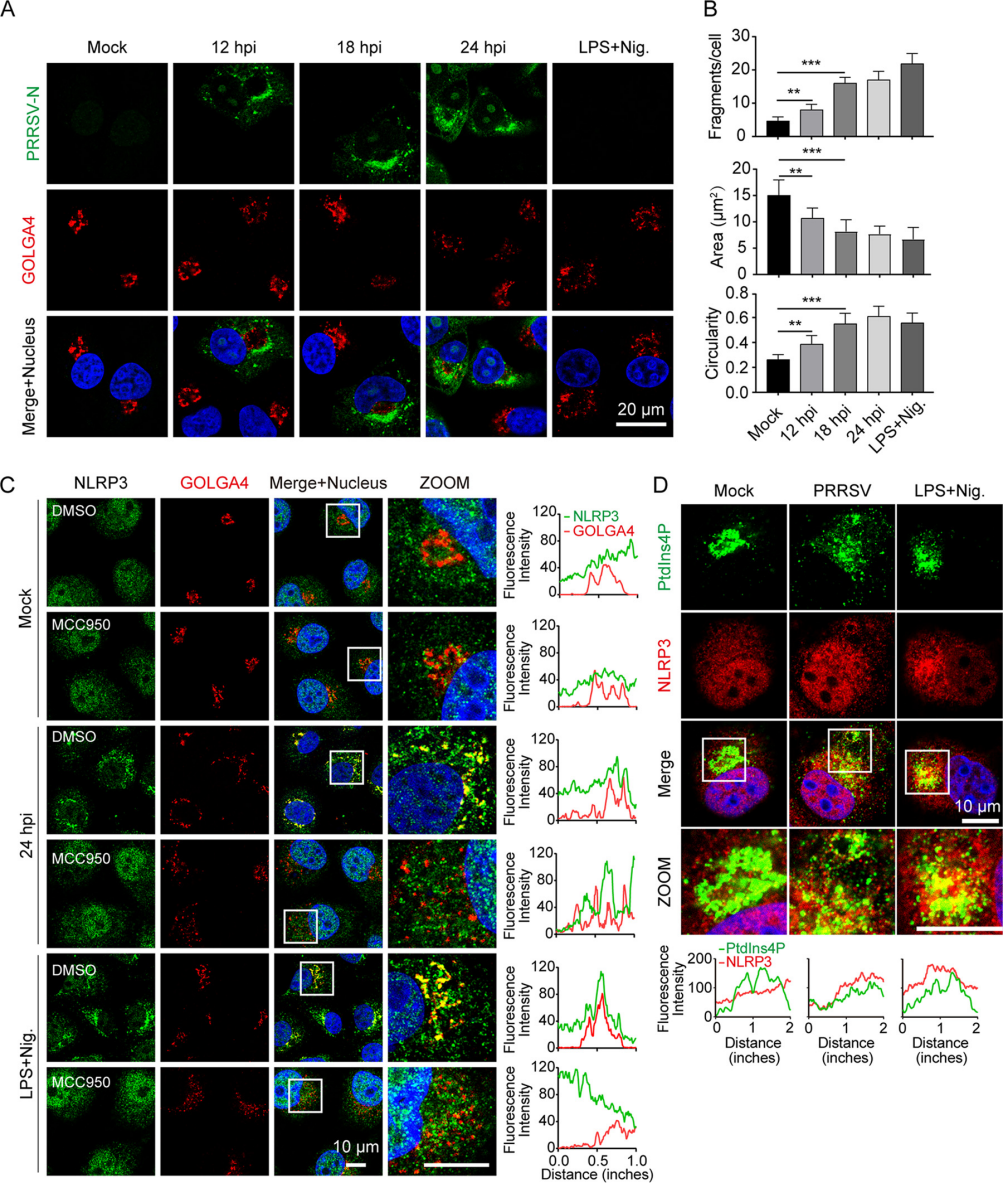
Phosphatidylinositol 4-phosphate (PtdIns4P) on dispersed trans-Golgi network mediates NLRP3 inflammasome activation during PRRSV infection. (A and B) Representative images showing that substantial trans-Golgi network (TGN) disassembly occurred in cells with PRRSV infection and nigericin stimulation, but not in mock-infected cells. Marc-145 cells were mock-infected or infected with PRRSV (MOI = 1) and fixed at the indicated time. As a positive control, cells were primed with LPS for 12 h, stimulated with nigericin (10 μM) for 45 min, and fixed. The trans-Golgi protein GOLGA4 (red) and PRRSV-N (green) were stained with specific antibodies. Nucleic acids were stained with DAPI (blue) (A). To quantify TGN disassembly, the numbers, areas, and circularities of TGN fragments for each cell were quantified from 200 randomly selected cells using ImageJ software for the data presented in panel A. Data represent the means ± SEM of ~200 cells for TGN markers from two independent experiments. (C) Immunofluorescence of cells labeled with NLRP3 and PRRSV-N antibodies. Marc-145 cell treatment was consistent with that shown in panel A, with or without MCC950. White boxes indicate zoomed areas. Colocalization quantifications were done using the Plot Profile plugin in ImageJ. (D) Marc-145 cells were treated as in panel A and stained with PtdIns4P and NLRP3 antibodies, followed by observation with confocal microscopy. Nuclei were counterstained with DAPI. White boxes mark the zoomed areas shown in the bottom subpanels. Intracellular co-localization analysis of PtdIns4P and NLRP3 was done using the Plot Profile plugin in ImageJ.

Due to the broad range and structural diversity of NLRP3 activators, there has been no detailed investigation of its direct interaction with any of these numerous agonists. Recent evidence reveals that NLRP3 recognizes diverse stimuli via the common mechanism of trans-Golgi network dispersion (22). We compared the architecture of the TGN apparatus (indicated by GOLGA4) in Marc-145 at various stages of PRRSV infection and found that it markedly affected the structure of this organelle to different degrees. Interestingly, tubulovesicular structures were present in PRRSV-uninfected (PRRSV-N negative) cells, whereas they were were completely dispersed into discrete vesicles in PRRSV-infected (PRRSV-N positive) and nigericin-treated cells (Fig. 5A). This finding strongly indicated that the changes in TGN morphology between the PRRSV-infected and uninfected cells was caused by viral infection. We next performed a detailed analysis and quantification of the dispersed TGN morphology using confocal imaging. This assessment revealed a marked increase in Golgi fragments in PRRSV-infected and nigericin-stimulated cells (Fig. 5B). Meanwhile, the area of the TGN fragments was reduced, and the circularity of TGN fragments increased, upon PRRSV infection and nigericin stimulation (Fig. 5B).

There was also significant variation in the distribution of NLRP3 between PRRSV-infected and uninfected cells. NLRP3 was diffused across the intracellular under uninfected conditions, while it formed multiple small puncta upon PRRSV infection and nigericin treatment (Fig. 5C), indicating that these puncta are the active form of NLRP3 (23). Moreover, after this stimulation and staining NLRP3 and GOLGA4, the TGN disassembled from tubulovesicular structures into discrete vesicles on which NLRP3 formed puncta (Fig. 5C). According to fluorescence intensity plot profile analysis, this finding revealed a striking colocalization of NLRP3 with GOLGA4 (Fig. 5C). However, in the presence of MCC950, NLRP3 puncta formation was blocked, and TGN dispersal was not affected after infection with PRRSV for 24 h or treatment with nigericin for 45 min (Fig. 5C), suggesting that dTGN formation may be a prerequisite for NLRP3 activation. In addition, we did not find a significant degree of overlap between NLRP3 and GOLGA4 staining under basal conditions, or in PRRSV-infected or nigericin-stimulated cells, in the presence of MCC950 (Fig. 5C). These results indicate that PRRSV infection leads to disassembly of the TGN, which is essential for NLRP3 aggregation and activation.

Prior studies have noted the importance of the dTGN for NLRP3 activation (23). The dTGN acts as a scaffold to recruit NLRP3 via phosphatidylinositol 4-phosphate (PtdIns4P), promoting the NLRP3 puncta formation on the dTGN in response to diverse NLRP3 activators (23). To test whether PRRSV infection mediates NLRP3 activation through a similar mechanism, we investigated the intracellular distribution changes of PtdIns4P during PRRSV infection and nigericin stimulation compared with that in mock treatment, and tested its co-localization with NLRP3 by confocal microscopy. After staining PtdIns4P and NLRP3, the PtdIns4P disassembled from a single perinuclear cluster (mock-infected cells) into discrete vesicles on which NLRP3 formed puncta during PRRSV infection and nigericin stimulation (Fig. 5D). As expected, colocalization of NLRP3 with PtdIns4P was not found in PRRSV-uninfected cells. Collectively, these results confirm that PRRSV infection leads to dTGN formation, which serves as a scaffold for NLRP3 inflammasome activation via PtdIns4P.

### Replication-transcription complex formed by PRRSV infection initiates pyroptosis.

**FIG 6:**
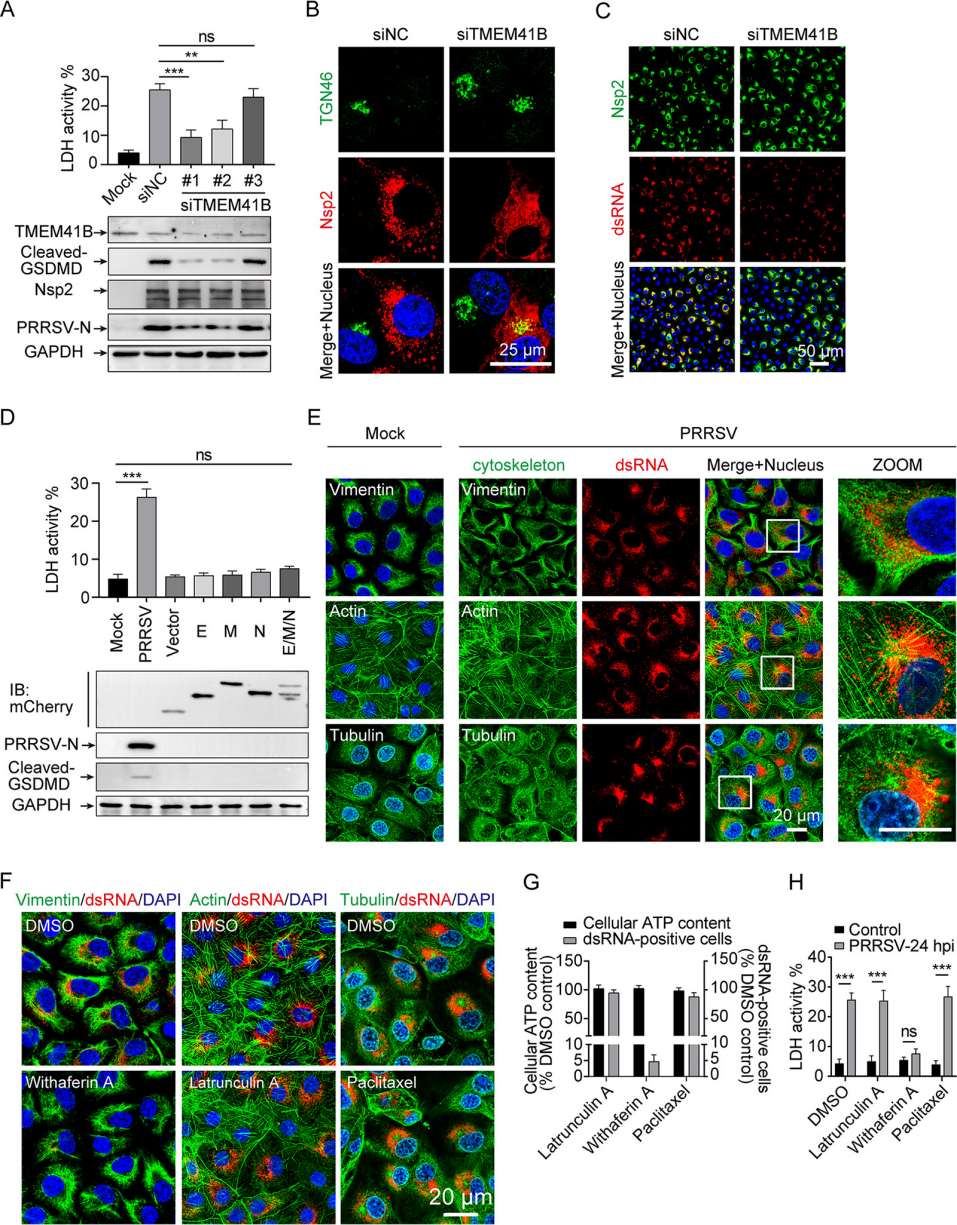
Replication-transcription complex (RTC) formed by PRRSV infection initial pyroptosis. (A to C) Marc-145 cells were infected with PRRSV (MOI = 1) for 24 h after TMEM41B knockdown. (A) PRRSV-nsp2, PRRSV-N, TMEM41B, cleaved GSDMD, and GAPDH proteins in the lysates were determined by Western blotting. Cell death was assessed using an LDH release assay. (B) Morphological changes in the TGN were observed by immunofluorescence. (C) Expression of PRRSV-nsp2 and dsRNA was analyzed by immunofluorescence. (D) Marc-145 cells were infected with PRRSV for 24 h or transfected with mCherry-tagged PRRSV structural proteins (envelope [E], membrane [M], and nucleocapsid [N]) for 24 h before being treated with LPS for another 12 h. Cells were collected and PRRSV-N, cleaved GSDMD, and GAPDH proteins in the lysates were detected using Western blot analysis. Cell death was assessed using an LDH release assay. (E) Marc-145 cells were infected with PRRSV for 24 h (MOI = 5), fixed, and stained with antibodies directed against dsRNA (the viral replication intermediate), α-tubulin, and vimentin. Actin filaments (green) were stained with phalloidin fluorescein isothiocyanate (FITC) reagent. Nuclei were stained with DAPI (blue). The regions in white boxes are magnified in the insets on the right. (F) Marc-145 cells were infected with PRRSV and treated with the cytoskeleton-altering compounds. Before the addition of withaferin A (5 μM), latrunculin A (1 μM), and paclitaxel (10 μM), the infection was allowed to progress for 2 h. After PRRSV infection for 24 h, cells were fixed, and dsRNA and the indicated cytoskeleton elements were detected by immunofluorescence microscopy using specific antibodies. Actin filaments were stained with phalloidin FITC reagent. (G and H) Cell viability after 22 h of treatment with the indicated compounds, as measured by intracellular ATP levels. Percentage of infected cells as determined by dsRNA staining of cells treated as in panel F. (H) Cell death was assessed using an LDH release assay. Data are representative of at least three independent experiments and shown as mean ± SEM. ns, not significant; **, *P* < 0.01; ***, *P* < 0.001 (one-way ANOVA).

The formation of replication-transcription complex is a vital stage in the replication of positive-stranded RNA viruses. To fully elucidate the mechanism underlying the observed TGN dispersion and pyroptosis signaling in PRRSV infection, we first inhibited PRRSV replication to investigate the involvement of RTC formation in TGN dispersion and pyroptosis activation in Marc-145 cells. Transmembrane protein 41B (TMEM41B) has been identified as a critical host factor for flavivirus and coronavirus RTC formation (33, 34). TMEM41B expression was downregulated with three small interference RNAs (siRNAs), respectively. Here, the effect of RTC suppression by TMEM41B knockdown was observed by Western blot assay, where TMEM41B knockdown significantly inhibited PRRSV-N production but there was no significant change in the expression of PRRSV nsp2, indicating that it had no obvious effects on viral nonstructural protein (nsp) translation, as this occurs before RTC formation (Fig. 6A). PRRSV nsp2 is a key protein that interacts with nsp3, nsp5, and host proteins to assemble into the RTC of PRRSV (35, 36). Moreover, TGN dispersion, GSDMD-NT production, and LDH release induced by PRRSV infection were attenuated by si-TMEM41B (Fig. 6A and B). A similar result was observed by immunofluorescence assay, where TMEM41B knockdown significantly inhibited PRRSV RTC formation (represented by the viral replication intermediate, dsRNA), while the expression of PRRSV nonstructural protein (nsp2) was not affected during PRRSV infection for 24 h (Fig. 6C), indicating that PRRSV RTC formation depends on the host protein TMEM41B. These results imply that inhibiting the formation of RTC maintains the TGN morphology and inhibits the occurrence of pyroptosis, and that PRRSV nonstructural proteins alone are not sufficient to induce pyroptosis.

The RTC is the site where *Arteriviridae* replicate viral genomes and produce plus-stranded subgenomic mRNAs (36). Subsequently, the subgenomic mRNAs are essential to express the structural protein genes which reside in the 3′-proximal quarter of the genome (5). According to the characteristics of PRRSV replication and viral protein translation, TMEM41B silencing attenuated pyroptosis caused by PRRSV infection due to hindering the formation of PRRSV RTC or the production of viral structural proteins. In combination with the structural proteins of other RNA viruses reported in the literature which caused NLRP3 inflammasome activation and pyroptosis (17, 18, 37), we investigated the roles of the envelope (E), matrix (M), and nucleocapsid (N) proteins of PRRSV in NLRP3 inflammasome activation and pyroptosis. However, GSDMD-NT was not detectable in the cell lysate, and LDH release in cell supernatants was not significantly altered by overexpression of these structural proteins (Fig. 6D). It is suggested that RTC generated by PRRSV infection, rather than by virus-derived protein translation, causes pyroptosis.

In addition to remodeling the cellular endomembrane system, the formation of virus RTC has been reported to be closely related to the cytoskeleton (38). To this end, we evaluated potential cytoskeleton alterations in PRRSV-infected cells and verified the influence of PRRSV RTC formation on pyroptosis from a cytoskeleton aspect. Using Marc-145 cells, we analyzed the alterations of different classes of cytoskeletal filaments during PRRSV infection for 24 h, including microtubules, microfilaments, and intermediate filaments. Compared with that in uninfected cells, in PRRSV-infected cells, no significant alterations were observed in microfilaments, and the alterations in microtubules were not closely related to dsRNA (Fig. 6E). Interestingly, dsRNA was surrounded by a cage-like structure formed by intermediate filaments in PRRSV-infected cells, indicating that intermediate filaments might serve to scaffold or segregate the PRRSV RTC compartment in space (Fig. 6E). To further confirm whether the cytoskeletal network remodeling promotes PRRSV RTC formation, we treated PRRSV-infected Marc-145 cells with pharmaceuticals which alter cytoskeleton dynamics. Compared with dimethyl sulfoxide (DMSO) treatment, the PRRSV RTC formation was robustly reduced by withaferin A, a compound that disrupts the intermediate filament network, whereas treatment with latrunculin A (a microfilament-disrupting agent) and paclitaxel (a microtubule stabilizer) had no effect (Fig. 6F). Notably, the concentrations of these three drugs did not cause cytotoxicity, as determined by quantification of intracellular ATP levels (Fig. 6G). Furthermore, after PRRSV infection, only withaferin A treatment had a strong inhibitory effect on the release of LDH, while neither latrunculin A nor paclitaxel did (Fig. 6H), suggesting an important role for PRRSV RTC formation in pyroptosis. In summary, PRRSV RTC formation, rather than PRRSV RNA or virus-encoded proteins, is the critical factor in PRRSV-induced pyroptosis; and interrupting RTC formation, either through the endomembrane system (si-TMEM41B) or the cytoskeleton (withaferin A), can partially rescue pyroptosis after PRRSV infection.

## DISCUSSION

**FIG 7:**
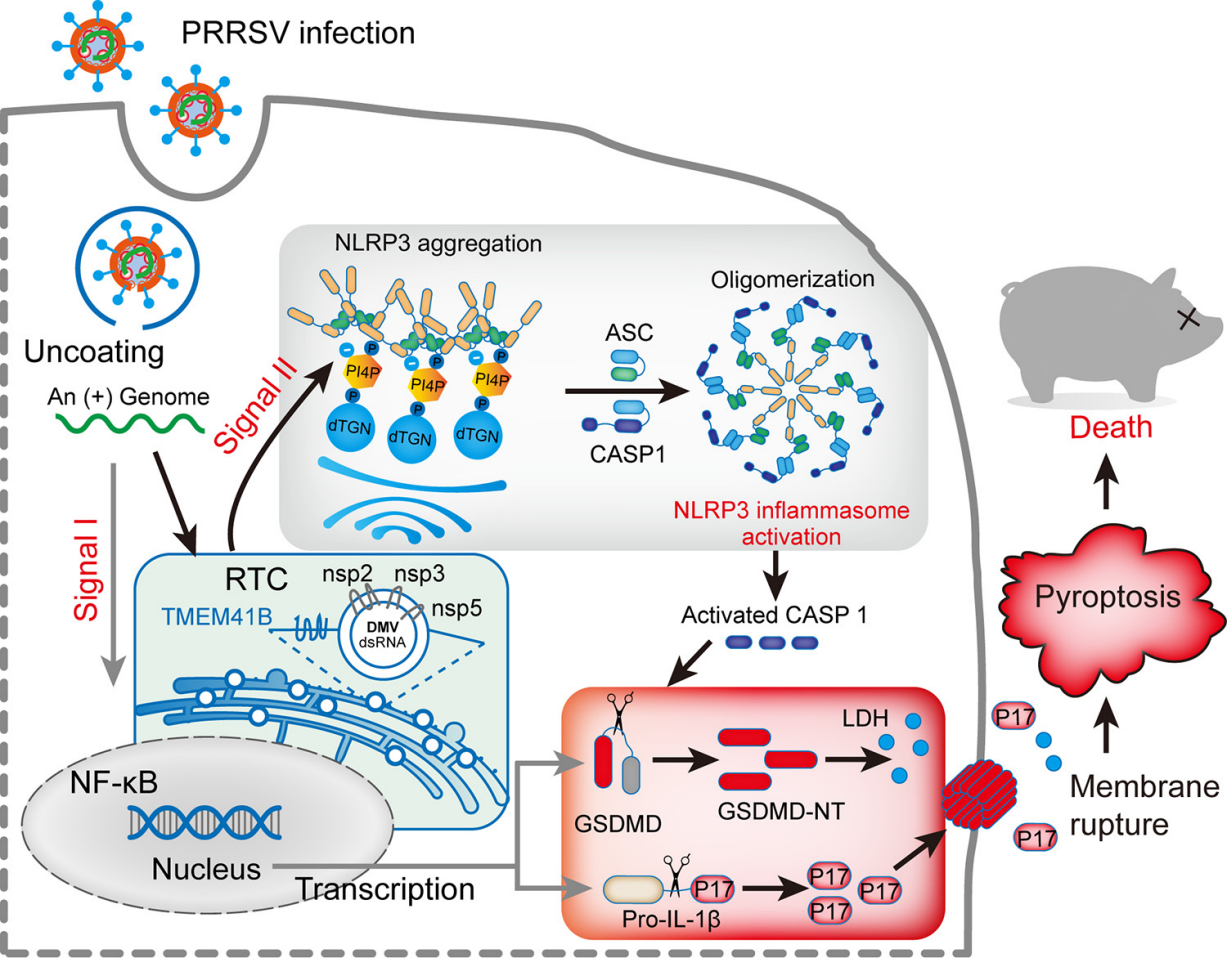
Schematic model: PRRSV infection induces NLRP3 inflammasome activation and pyroptosis. Signal I (priming; left side) is provided by PRRSV infection, leading to the transcriptional upregulation of NLRP3 inflammasome components and pro-IL-1β. Signal II (activation; right side) is provided by the PRRSV RTC-induced dispersed *trans*-Golgi network (dTGN), which activates NLRP3, followed by the recruitment of ASC and CASP1 to form the NLRP3 inflammasome complex. Formation of the NLRP3 inflammasome activates CASP1, which in turn cleaves pro-IL-1β and GSDMD. After cleavage, the N terminus of GSDMD (GSDMD-NT) inserts into the membrane, forming pores and inducing pyroptosis.

Our results show that PRRSV, a positive-stranded RNA virus, activates the NLRP3 inflammasome and GSDMD-dependent pyroptosis through TGN dispersion induced by viral RTC formation, resulting in increased secretion of IL-1β and LDH. Given that various RNA viruses have been demonstrated to induce pyroptosis through the NLRP3 inflammasome (30, 39, 40), our finding that NLRP3 inflammasome activation contributes to PRRSV-induced IL-1β secretion and pyroptosis both *in vitro* and *in vivo* is remarkable. In this study, a novel mechanism by which PRRSV infection activates the NLRP3 inflammasome to facilitate IL-1β maturation and pyroptosis is revealed (Fig. 7). In addition, we show that PRRSV infection induces the disassembly of TGN, and that the dTGN then acts as a scaffold to recruit NLRP3 via PtdIns4P, promoting NLRP3 activation. Furthermore, from the perspective of the virus, PRRSV RTC is surrounded by a network of intermediate filaments and is identified as a key pyroptosis activator. Thus, we reveal a mechanism by which PRRSV induces host inflammatory and pyroptosis.

Due to the tremendous economic losses caused by PRRSV infections, sustained efforts dedicated to understanding PRRSV pathogenesis have been made since its identification (4). On the one hand, PRRSV vaccines have been developed and licensed for commercial use. However, the constantly evolving PRRSV strains and recombination between vaccine and wild-type strains results in inadequate vaccine efficiency and contributes to the high prevalence of PRRSV infection in swine herds (4, 41). On the other hand, PRRSV entry blockers, targeting host factors that mediate PRRSV infections, show promise for combating PRRSV infection. For example, knocking out CD163, the major receptor that mediates viral internalization and disassembly, protects pigs from PRRSV infection (42, 43). However, it is still far from being used in agriculture.

It appears that directly targeting the host factors which mediate PRRSV infections by small molecules might be a potential approach, based on the increasing evidence to further unmask cellular and molecular mechanisms. In our study, NLRP3 mediated pyroptosis of PAM upon PRRSV infection, and inhibiting NLRP3 activity by introducing MCC950 significantly relieved cell death. MCC950 is the most potent and specific NLRP3 inhibitor and shows therapeutic efficacy against NLRP3 activation in a variety of disease models (31). In addition, the formation of RTC is a critical event upstream of NLRP3 inflammasome activation, and blocking PRRSV RTC formation by affecting the function of TMEM41B and vimentin might be an alternative approach against PRRSV infection. In our study, siTMEM41B and withaferin A showed potential to prevent PRRSV infection. Therefore, small molecules targeting the host factors which mediate PRRSV infection deserve further investigations to develop potential therapeutic strategies.

PRRS manifests differently in pigs at different stages of development. However, severe respiratory distress caused by lesions in the porcine lungs is a common symptom, especially in fatal cases, and it has been proposed that respiratory distress plays a central role in the pathogenesis of PRRSV infection (6). Elevated pro-inflammatory cytokines and migration of inflammatory cells to lung tissues are the major contributors to the pathogenesis of most acute and chronic lung injuries (10, 44). Severe acute respiratory syndrome coronavirus (SARS-CoV), Middle East respiratory syndrome coronavirus (MERS-CoV), and avian influenza A H5N1 virus infection have demonstrated the high mortality due to the complication of acute respiratory distress syndrome (ARDS), which is likely to develop from acute lung injury (45, 46). During these respiratory virus infections, inflammatory responses mediated by alveolar macrophages have been reported to be the critical factor in inflammation-associated lung damage (44–47). There appears to be a correlation between the severity of clinical symptoms, pro-inflammatory cytokine expression, and inflammatory responses.

Pyroptosis represents a type of programmed cell death that is highly inflammatory and distinct from apoptosis or other types of cell death (13). GSDMD is a critical pyroptotic effector that is cleaved by CASP1 to generate the pore-forming p30 subunit, which disrupts cell membranes (24). During commonly pyroptotic infections, such as with HIV, Zika virus, and norovirus, IL-β maturation, LDH secretion, and GSDMD cleavage are intrinsic to the infected, dying cell (15, 39, 48). Several reports have shown that PRRSV infection causes IL-1β secretion in infected piglets and cells, and that siNLRP3 and siASC attenuate IL-1β secretion in infected PAMs, suggesting that the increase in pro-inflammatory cytokines induced by PRRSV infection is due to activation of the NLRP3 inflammasome (49, 50). Our work extends existing investigations and reveals that the formation of PRRSV RTC induces TGN dispersion and activates the NLRP3 inflammasome, which in turn leads to the cleavage of GSDMD and pyroptotic cell death. In such a context, our study facilitates understanding of how PRRSV causes acute lung injury and induces pro-inflammatory cytokine secretion.

Due to the vast number and structural diversity of NLRP3 activators, it is believed that NLRP3 does not directly interact with numerous activators, but rather senses a common intracellular event induced by these activators. Recently, it has been shown that a large variety of NLRP3 activators lead to disassembly of the TGN, and a strong relationship between Golgi dispersion and NLRP3 inflammasome activation has emerged (23). The dispersed TGN serves as a scaffold to recruit NLRP3 via PtdIns4P, promoting NLRP3 aggregation into multiple puncta and interaction with adaptor protein ASC, thereby initiating the downstream signaling cascade (23). However, this mechanism has not been demonstrated for the NLRP3 inflammasome activation caused by pathogen infection; we first verified it in the PRRSV infection. In our study, we demonstrated that PRRSV infection causes robust disassembly of the TGN and NLRP3 aggregation, and that NLRP3 co-localizes with GOLGA4 (TGN marker) and PtdIns4P after PRRSV infection. Actually, according to previous studies, pathological processes such as Alzheimer’s disease result in fragmentation of the Golgi apparatus, and infection with HCV and human rhinovirus (HRV), or intracellular bacteria such as *Shigella*, can also perturb Golgi architecture dispersal to varying degrees (51–53), and all of these cause NLRP3 inflammasome activation (18, 54–56). Besides this, some other studies have suggested that the Golgi apparatus acts as not only a critical sorting organelle but also as a platform for receiving and transmitting signals, thereby serving as a bridge to facilitate multiple signal transductions (22, 23, 57). It has been found that intracellular pathogens disrupt the Golgi apparatus to target its important factors, including Golgi lipid composition and immunity-related GTPase M (52, 58). Recent reports suggest that the pathogen effector proteins IpaJ and RARP2 are involved in Golgi apparatus fragmentation in Shigella flexneri- and Rickettsia rickettsii-infected cells, respectively (58, 59). However, during PRRSV infection, the cellular targets and effector proteins which attempt to disperse TGN remain unknown. Further investigations to unravel the complexity of Golgi apparatus structure and function may aid in better understanding the mechanisms of PRRSV infections.

Various viral factors, including viral genomic RNA, viral RNA transcripts, and protein production, have been reported to activate the NLRP3 inflammasome (15, 60, 61). In order to reveal the viral factors by which PRRSV activates the NLRP3 inflammasome, the effects of these factors were determined. Our study demonstrated that TGN dispersion induced by PRRSV RTC formation is essential for the activation of NLRP3 inflammasome. Our results differ from a those of a previous report which indicated NLRP3 inflammasome activation through DDX19A that recognized PRRSV RNA, *in vitro*-synthesized 5′ untranslated region (UTR), 3′ UTR, and nsp7b transcripts in PAMs (28). This controversial result could be referred to for the advancement of other viral infection investigations. In terms of HCV, although transcriptional induction of genes encoding pro-IL-1β and NLRP3 is activated through TLR7 that recognizes viral RNA, the NLRP3 inflammasome activation is caused by HCV core protein (18, 62). A similar finding was also reported by Muruve et al. (63), who showed that treatment of cells with poly(I:C) failed to elicit NLRP3 inflammasome activation, indicating that viral RNA cannot activate the NLRP3 on its own.

Recent studies have found that TMEM41B is a critical host factor for pan-coronavirus RTC formation, as well as in several flaviviruses, suggesting that TMEM41B is a broad-spectrum RNA virus RTC formation liability (33, 64). Interestingly, TMEM41B contributes to the formation of PRRSV RTC, which has been proven in this study. One possible explanation is that TMEM41B is recruited to PRRSV RTC to facilitate intracellular membrane remodeling, similar to how flavivirus RTC are formed (33). Considering that PRRSV belongs to the family *Arteriviridae* in the order *Nidovirales*, the production of structural proteins includes a discontinuous step to produce subgenomic mRNAs, which are generated in the RTC (36, 65). Therefore, the envelope (E), matrix (M), and nucleocapsid (N) proteins of PRRSV were overexpressed in Marc-145 cells. However, they exhibited no effect on promoting pyroptosis.

The hypothesis that the formation of RTC depends on cytoskeleton alterations was supported by the fact that pyroptosis can be prevented through interruption of intermediate filaments by withaferin A treatment. These results corroborate those of Cortese et al. (38), who suggested that intermediate filaments serve to scaffold or confine the SARS-CoV-2 RTC as a critical host factor contributing to SARS-CoV-2 replication. Although the function of the PRRSV RTC has rarely been reported previously, it has been unmasked in other viral infections. The RTC formed by equine arteritis virus, which also belonging to the *Arteriviridae*, is thought to create an optimal microenvironment conducive to viral RNA synthesis by allowing the enrichment of viral replicative proteins and relevant host factors, as well as protecting viral replication intermediates from degradation and detection by the innate immune system (36). Here, we revealed a novel function of PRRSV RTC in the regulation of PRRSV-induced pyroptosis, and the mechanisms of PRRSV RTC formation warrant further investigation.

Although PRRSV has been demonstrated to induce the non-inflammatory response of apoptosis in cells and pigs, the exact cell death mechanism responsible for the inflammatory responses that may determine the pathogenesis of PRRSV remains poorly understood (66, 67). Our study extends existing findings by highlighting the activation of NLRP3 inflammasome and the occurrence of pyroptosis in response to PRRSV infection, which result in the extracellular release of proinflammatory cytokines, such as IL-1β. IL-1β is an important participant in the regulation of immune cell migration, particularly that of monocytes and neutrophils, to remove infectious agents (15). However, these migrated immune cells also contribute to excessive inflammation, which leads to pneumonia (30). Thus, the inflammatory response mechanism of the host defense is crucial for understanding the pathogenesis of PRRSV. In addition, until now, we have not been able to directly address whether pyroptosis plays a protective or a detrimental role against PRRSV infection in pigs. Nonetheless, these results collectively show that pyroptosis promotes pathogen elimination but is also associated with tissue damage and a chronic inflammatory response (68, 69). Instead of inducing classic triggers which promote either apoptosis or pyroptosis, microbial infections always prefer the cross talk between apoptosis and pyroptosis (40, 70). According to current research, there may be cross talk of programmed cell death pathways during PRRSV infection, even if the specific, central molecular mechanisms need further investigation to determine the pathways of programmed cell death.

Overall, this study demonstrates that PRRSV infection can trigger pyroptosis via NLRP3 inflammasome activation *in vitro* and *in vivo*, paving the way for a more detailed understanding of PRRSV-induced inflammatory responses and cell death pathways. This study determined that PRRSV RTC formation-induced dispersed TGN serves as a scaffold for NLRP3 recruitment via PtdIns4P, promoting NLRP3 activation. Considering the common mechanism by which different NLRP3 activators cause NLRP3 inflammasome activation via PtdIns4P on dTGN (23), it would be interesting to investigate whether other viruses that are known to cause TGN disassembly into dTGN, such as HCV and HRV (52, 53), trigger NLRP3 inflammasome activation through a similar mechanism. Moreover, our research discovered that PRRSV RTC formation is a key regulator of NLRP3 inflammasome activation. Many virus infections, particularly those of positive-strand RNA viruses, are known to form RTC in the cytoplasm, (71, 72). Despite the fact that the RTCs formed by HCV, HRV, and SARS-CoV-2 are morphologically distinct (38, 71, 72), their infections have been reported to activate NLRP3 inflammasome (18, 55, 73). Thus, we suspect that viral RTC, in addition to acting as a physical scaffold to concentrate viral components and increase replication efficiency, may be an indirect target for cellular stress sensors. It should also be investigated whether the formation of RTC by viral infection is a common mechanism of NLRP3 activation. Such investigations which elucidate the relevance of viral RTC formation and TGN dispersion in the activation of innate immune receptors will help develop anti-inflammation strategies and treatments for the prevention of severe immunopathology induced by a highly pathogenic virus.

## MATERIALS AND METHODS

### Ethics statement.

The license number for this research protocol is 2021c003, which was approved by The Laboratory Animal Ethical Committee of South China Agricultural University. Animal experiments were carried out according to the Guide for the Care and Use of Laboratory Animals and Laboratory Animal-Requirements of Environment and Housing Facilities (GB14925-2010/XG1-2011, National Laboratory Animal Standardization Technical Committee), which ensures humane and ethical treatment of animals.

### Cells and viruses.

PAMs were prepared from the lung lavage fluid of 4-to 6-week-old healthy euthanasia piglets free of PRRSV, as previously described (74). PAMs were cultured in RPMI 1640 medium (Gibco, NY) supplemented with 10% (vol/vol) heat-inactivated fetal bovine serum (FBS) (Gibco), 100 U/mL penicillin, and 100 μg/mL streptomycin (Gibco). African green monkey embryonic kidney cell line Marc-145 (China Center for Type Culture Collection, China) was cultured in Dulbecco’s modified Eagle’s medium (DMEM; Corning, Corning, NY) supplemented with 10% (vol/vol) FBS, penicillin, and streptomycin as mentioned above. It was free of mycoplasma contamination, based on the results of Mycoscan Mycoplasma Detection Kit (Cellcook, Guangzhou, China), and regularly maintained with Normocin (ant-nr-2, InvivoGen, San Diego, CA). All cells were cultured at 37°C in a humidified atmosphere with 5% (vol/vol) CO_2_. The PRRSV genotype 2 strains JXA1 (GenBank accession no. EF112445.1) for *in vitro* experiments and GDBY1 (GenBank accession no. GQ374442.1) for *in vivo* experiments were provided by Guihong Zhang (South China Agricultural University, Guangzhou, China) and Yongchang Cao (Sun Yat-sen University, Guangzhou, China), respectively. These PRRSV strains were propagated in Marc-145 cells and stored at −80°C, and viral titers were quantified by a microtitration infectivity assay to 50% tissue culture infective dose (74).

### Challenge with PRRSV strain GDBY1 in pigs.

Eighteen 4-week-old PRRSV negative pigs were randomly divided into two groups, the GDBY1-infected group (*n* = 12) and the mock-infected control group (*n* = 6). Each group was then housed in a separate isolation room with suitable temperature and humidity and individual ventilation, where water and food were available *ad libitum*. After 1 week of adaptive breeding, viral inoculation was conducted by nasal intubation drip (2 mL: 4.4 × 10^5^ TCID_50_/mL) and intramuscular injection (2 mL: 4.4 × 10^5^ TCID_50_/mL) with a highly pathogenic PRRSV (HP-RRSV) strain, GDBY1 (75). The pigs in the mock-infected control group were inoculated with the same dosages of Marc-145 cell culture supernatant, DMEM, in the same way. At the 7-, 14-, and 21-days postinfection (dpi), we took 2 and 3 pigs from the control and the GDBY1-infected groups, respectively, which were humanely euthanized via pentobarbital overdose. For the pigs which died during the course of the PRRSV challenge, we took pictures and tissue samples immediately. All surviving pigs were slaughtered at 21 dpi. Lung tissue was used for hematoxylin and eosin (H&E) staining, immunohistochemistry, immunoblotting, and real-time quantitative reverse transcriptase PCR (qRT-PCR). Bronchoalveolar lavage fluid (BALF) and PAMs were collected as previously described for the detection of pro-inflammatory cytokines (10, 44).

### Antibodies.

Mouse monoclonal antibody (MAb) to PRRSV nucleocapsid (N) protein (JN0401) (American type specific) was purchased from J&T (Shanghai, China). Rabbit anti-PRRSV N polyclonal antibody (pAb) (GTX129270) and rabbit anti-vimentin pAb (GTX100619) were purchased from GeneTex (Irvine, CA). Mouse anti-dsRNA (J2) MAb was purchased from Scicons (Budapest, Hungary). Rabbit and mouse antibodies to PRRSV nsp2 were provided by Hanchun Yang from China Agricultural University. Rabbit anti-NLRP3 pAb (bs-10021R), rabbit anti-caspase-1 p20 pAb (bs-10743R), and rabbit anti-HMGB1 pAb (bs-20633R) were purchased from Bioss (Beijing, China). Mouse anti-ASC MAb (sc-514414) was purchased from Santa Cruz Biotechnology (Dallas, TX). Rabbit anti-gasdermin D pAb (ab155233) and mouse anti-58K Golgi MAb (ab27043) were purchased from Abcam (Cambridge, United Kingdom). Mouse MAb against human p230 trans Golgi MAb (GOLGA4) (611280) was obtained from BD Biosciences (East Rutherford, NJ). Rabbit MAb GAPDH (glyceraldehyde-3-phosphate dehydrogenase; 2118s), cleaved caspase-1 (Asp297) (4199s), and cleaved gasdermin D (Asp275) (36425s) were purchased from Cell Signaling Technology (CST, Danvers, MA). Mouse anti-PI4P antibody (z-p004) was purchased from Echelon Biosciences Inc. (Echelon Biosciences, Salt Lake City, UT). Anti-porcine IL-1β antibody (AF681) was purchased from R&D Systems (R&D, Minneapolis, MN). Goat anti-rabbit IgG, horseradish peroxidase (HRP)-linked antibody (7074s) and horse anti-mouse IgG, HRP-linked antibody (7076s) were purchased from Cell Signaling Technology. Rabbit anti-goat IgG, HRP-linked antibody (BS30503) was purchased from Bioworld Technology (Bioworld, Shanghai, China). Except for Alexa Fluor 488 goat anti-mouse IgM (A21042), which was purchased from Invitrogen, the other Alexa Fluor secondary antibodies were purchased from Cell Signaling Technology.

### Reagents.

Lipopolysaccharides (LPS) (L2630) were obtained from Sigma (Sigma-Aldrich, St. Louis, MO). Cycloheximide (S7418), nigericin sodium salt (S6653), and MCC950 sodium salt (S7809) were obtained from Selleck (Houston, TX). Withaferin A (M7490), latrunculin A (M6896), and paclitaxel (M1970) were obtained from Abmole (Houston, TX). Z-YVAD-FMK (ab141388) and phalloidin fluorescein isothiocyanate (FITC) reagent (ab235137) were obtained from Abcam. 7-Actinomycin D (7-AAD, A1310) and disuccinimidyl suberate (DSS) (21655) were obtained from Invitrogen.

### Western blotting.

Cells were washed once in phosphate-buffered saline (PBS) and lysed in cell lysis buffer (P0013, Beyotime, Shanggai, China) containing phenylmethylsulfonyl fluoride (PMSF; ST506, Beyotime). Cell lysates were clarified by centrifugation, separated by SDS-PAGE, and semi-dry transferred to a polyvinyl difluoride (PVDF) membrane (Millipore, Darmstadt, Germany). Membranes were blocked in blocking buffer (P0252, Beyotime) for 30 min at room temperature before incubation with the indicated antibodies overnight at 4°C. After three washings with Tris-buffered saline containing 0.1% Tween 20, the membranes were incubated with the corresponding secondary antibodies for 1 h at room temperature. Protein bands were visualized by enhanced chemiluminescence reagent, according to the manufacturer’s instructions (Fdbio Science, Hangzhou, China).

To detect the secretion of IL-1β p17 and CASP1 p20, the PAM cell culture supernatants were concentrated by TCA (trichloroacetic acid) protein precipitation kit (C510011, Sangon Biotech, Shanghai, China), according to the manufacturer’s instructions. The obtained samples were loaded for SDS-PAGE. After electrophoresis, the indicated IL-1β p17 and caspase-1 p20 were detected by Western blotting.

### Co-immunoprecipitation assays.

In order to detect NLRP3 inflammasome formation by immunoprecipitation, during PRRSV infection, we referenced the procedures of previous reports, LPS-primed PAMs, and treated with 10 μM nigericin for 1 h as the positive control. Cells were then lysed in cell lysis buffer (P0013, Beyotime) supplemented with PMSF and EDTA-free protease inhibitor cocktail (78425, Invitrogen). Next, 5% of the lysates were collected as whole-cell lysates mixed with SDS loading buffer and heated at 100°C for 10 min. Immunoprecipitation was performed according to the manufacturer’s instructions in the Dynabeads protein G immunoprecipitation kit (10007D, Invitrogen). The lysates from PAMs were immunoprecipitated with anti-ASC antibody (sc-5144414, Santa Cruz) or mouse MAb IgG1 isotype control (5415, CST) followed by Dynabeads magnetic beads for 1 h at room temperature. After washing three times with washing buffer, bound proteins were eluted from the beads and analyzed by immunoblot for NLRP3, ASC, or caspase-1.

### Immunofluorescence assay.

Marc-145 cells or PAMs were plated onto 15-mm cover glass-bottomed petri dishes (801002, Nest, Wuxi, China) and then used for stimulation. For immunostaining, cells were fixed with 4% paraformaldehyde for 15 min at room temperature and permeabilized with 0.1% Triton X-100 in PBS for 5 min. Before incubation with primary antibodies followed by Alexa Fluor secondary antibodies, nonspecific antibody sites were blocked with blocking buffer (P0260, Beyotime) for 30 min at room temperature. Primary antibodies were incubated overnight at 4°C, and secondary antibodies were incubated for 1 h while rocking in the dark. Nuclei were stained with DAPI (4’,6-diamidino-2-phenylindole) in mounting with prolong gold antifade reagent (P36934, Invitrogen). In addition, we also used 0.1% saponin in place of 0.1% Triton X-100 in the permeabilization step and 5% BSA in place of blocking buffer to better preserve the dTGN structures and observe the PI4P in fixed cells. Fluorescent images of fixed cells were taken with a confocal laser scanning microscope (TCS-SP5, Leica, Germany).

To quantify the cells with phenotypes of interest, 25 non-overlapping full field images were taken randomly throughout the slide of each sample. Only the DAPI channel was used to select the image field, and image acquisition parameters remained constant during imaging to avoid bias in the selection of cells with a specific phenotype. Image J software was used for quantitative confocal image analysis. The fluorescence intensity of TGN with or without PRRSV infection was determined by background subtraction and using a fixed threshold. For quantification of TGN disassembly, the number of cells with the phenotype of interest was recorded from 100 cells and this process was repeated three times. Given the mitotic Golgi disassembly, cells undergoing mitosis were excluded from the quantifications above. Next, the TGN fragment particle numbers and areas were quantified using the Analyze Particle plugin in ImageJ (https://imagej.nih.gov/ij). After a freehand selection option in ImageJ software to outline the TGN based on GOLGA4 staining, circularity index values were calculated according to the format of the ImageJ circularity plugin, in which circularity = 4 π (area/perimeter^2^) (52). The fluorescence intensity plot profile analysis in ImageJ was used for co-location analysis.

### Flow cytometry.

CASP1 activation was determined using the FLICA pyroptosis detection kit for CASP1 (9146, Immunochemistry Technologies, Bloomington, MN) according to the manufacturer’s guidelines, with slight modifications. In addition to the indicated time points of PRRSV infection PAMs, the LPS-primed PAMs treated with 10 μM nigericin for 2 h were used as the positive control. PAMs were incubated with 2.5 μM FAM-YVAD-FMK FLICA for 1 h in Opti-MEM. Subsequently, cells were incubated with 7-AAD (A1310, Invitrogen) for 10 min prior to flow cytometry analysis fluorescence.

### Inflammasome activation.

To activate the NLRP3 inflammasome, PAMs or Marc-145 cells were treated with 100 ng/mL LPS for 12 h and then stimulated with 10 μM nigericin for 2 h. To better observe the TGN dispersed, Marc-145 cells were primed with LPS and incubated with 10 μM nigericin for 45 min as the positive control.

### ASC oligomerization detection.

Unlike cells from different time points of PRRSV infection, LPS-primed PAMs were treated with 10 μM nigericin for 1 h as the positive control. For ASC oligomer cross-linking, we adopted the procedures of previous reports (31). Briefly, cells were lysed using the cell lysis buffer (P0013, Beyotime) supplemented with PMSF and EDTA-free protease inhibitor cocktail (78425, Invitrogen). Lysates were pipetted, vortexed, and centrifuged at 6,000 × *g* at 4°C for 15 min. The supernatants of lysates were mixed with SDS loading buffer for Western blotting with the indicated antibodies. The pellets fractions were washed with PBS, re-suspended in 200 μL cell lysis buffer, and cross-linked using fresh DSS (final 2 mM) at room temperature for 30 min with rotation. Cross-linked pellets were collected after being centrifuged, and dissolved in 20 μL of 1× SDS loading buffer for Western blotting.

### Isolation of PRRSV RNA and transfection.

The acquisition of PRRSV virions was described previously (28). PRRSV genomic RNAs, PRRSV infected RNAs, and PRRSV uninfected RNAs were isolated from virions and Marc-145 cells infected or uninfected with PRRSV at 24 h postinfection using TRIzol reagent (R4801, Magen, Guangzhou, China), respectively. The concentration of RNA obtained was measured uusing NanoDrop technology (Thermo Fisher Scientific, Waltham, MA). Marc-145 cells were transfected with 10 μg total RNA from vRNA, iRNA, and uRNA using Lipofectamine 3000 (L3000015, Invitrogen). After 24 h posttransfection, cell-free supernatants, total RNA, and proteins were collected and used for enzyme-linked immunosorbent assay (ELISA), qPCR, and Western blot experiments, respectively.

### Quantitative real-time PCR.

Total RNA was extracted from cells using TRIzol reagent (R4801, Magen, China) according to the manufacturer’s instructions. For real-time quantitative reverse transcriptase PCR (qRT-PCR) analysis, cDNA was generated from total RNA (1 μg) with a Reverse Transcription System (A3500, Promega, Madison, WI) in a 10-μL reaction volume, following the manufacturer’s protocol, and analyzed by quantitative real-time PCR using the SYBR Green qPCR Mix (A301, GenStar, China) on a LightCycler 480 real-time PCR system (LC480, Roche, Switzerland). Relative expression of target genes was calculated using the threshold cycle (2^−ΔΔCT^) method and normalized to HPRT1 expression (74, 76). The real-time PCR primers used above are as follows:

IL-1β Fw: 5′-TCCAGCCAGTCTTCATTGTT-3′;

IL-1β Rv: 5′-GATGACAGACACCATCTGCCT-3′;

PRRSV-N Fw: 5′-AAAACCAGTCCAGAGGCAAG-3′;

PRRSV-N Rv: 5′-CGGATCAGACGCACAGTATG-3′;

HRPT1 Fw: 5′-TGGAAAGAATGTCTTGATTGTTGAAG-3′;

HRPT1 Rv: 5′-ATCTTTGGATTATGCTGCTTGACC-3′.

### RNA interference.

The small interference RNAs (siRNAs) against TMEM41B and siRNA-negative control (NC) were obtained from GenePharma (Shanghai, China) and transfected using RNAiMAX (13778150, Invitrogen, Waltham, MA) according to the manufacturer’s instructions for 24 h. Indicated siRNAs used in this study are as follows:

siNC: 5′-UUCUCCGAACGUGUCACGUTT-3′;

TMEM41B siRNA 1: 5′-GGAUGAUGCCAAGGCUCUATT-3′;

TMEM41B siRNA 2: 5′-GCUAUUCCAGGCUCUAUAUTT-3′;

TMEM41B siRNA 3: 5′-GGAGACCAGUUGUAUACAATT-3′.

### Plasmid constructions.

The cDNAs encoding PRRSV (JXA1 strain) E, M, and N proteins were obtained by reverse transcription and PCR of total RNA from PRRSV-infected Marc-145 cells, and cloned into pmCherry-N1 vector (632523, TaKaRa Bio, Japan) with an N-terminal mCherry tag, respectively.

### Cytotoxicity assay and IL-1β ELISA.

PAMs or Marc-145 cells were treated as indicated. Cell death was measured through LDH release using a CytoTox 96 Non-Radioactive Cytotoxicity Assay kit (G1780, Promega, WI) according to the manufacturer’s instructions. LDH activity in cell culture supernatants was detected using the kit reagents and calculated according to the 100% LDH release control from lysed cells. The concentrations of secreted IL-1β in the PAMs culture supernatant, pig serum, and bronchoalveolar lavage fluid were determined by ELISA using a Swine IL-1β ELISA kit (CSE0013, 4A Biotech, Beijing, China) according to the manufacturer’s instructions.

### Hematoxylin and eosin staining and immunohistochemistry.

Piglet lung tissues were isolated and fixed in 10% formaldehyde for 24 h at room temperature. Fixed tissues were dehydrated, embedded in paraffin, and cut in 8-μm sections. For histopathology, sections were rehydrated and stained with hematoxylin and eosin (C0105S, Beyotime) according to the manufacturer’s instructions.

For immunohistochemistry, tissue sections from pig lungs were placed on silane-coated slides, deparaffinized, and rehydrated using decreasing concentrations of ethanol. The sections were then treated with cell and tissue staining kit reagents (CTS005, CTS002, R&D), according to the manufacturer’s instructions, after being incubated with 10 mM citrate buffer for heat-induced antigen retrieval. The following primary antibodies were used at the respective concentrations: rabbit anti-PRRSV N (1:200, GTX129270, GeneTex), rabbit anti-cleaved CASP1 (Asp297) (1:200, 4199s, CST), mouse anti-ASC (1:100, sc-514414, Santa Cruz), and rabbit anti-NLRP3 (1:200, bs-10021R, Bioss).

### Statistical analysis.

All experiments were performed with at least three independent replicates with similar results, and image data shown are representative of at least three randomly selected fields. Data were analyzed for statistical significance by Student’s *t* test or one-way analysis of variance (ANOVA). Means were illustrated using histograms by GraphPad Prism v8.0, with error bars representing standard error of the mean (SEM) in all graphs; values of *P* < 0.05 were considered statistically significant.
